# Milkshake Acutely Stimulates Dopamine Release in Ventral and Dorsal Striatum in Healthy-Weight Individuals and Patients with Severe Obesity Undergoing Bariatric Surgery: A Pilot Study

**DOI:** 10.3390/nu15122671

**Published:** 2023-06-08

**Authors:** Susan Carnell, Kimberley E. Steele, Gita Thapaliya, Hiroto Kuwubara, Anahys Aghababian, Afroditi Papantoni, Ayon Nandi, James R. Brašić, Timothy H. Moran, Dean F. Wong

**Affiliations:** 1 Division of Child and Adolescent Psychiatry, Department of Psychiatry and Behavioral Sciences, Johns Hopkins University School of Medicine, Baltimore, MD 21287, USA; gthapal2@jhmi.edu (G.T.); aaghaba1@jhmi.edu (A.A.); afroditi@live.unc.edu (A.P.); 2 Johns Hopkins Center for Bariatric Surgery, Department of Surgery, Johns Hopkins University School of Medicine, Baltimore, MD 21224, USA; steele.kimmd@outlook.com; 3 Section of High Resolution Brain Positron Emission Tomography Imaging, Division of Nuclear Medicine and Molecular Imaging, The Russell H. Morgan Department of Radiology and Radiological Science, Johns Hopkins University School of Medicine, Baltimore, MD 21287, USA; hkuwaba1@jhmi.edu (H.K.); anandi1@jhmi.edu (A.N.); jbrasic1@jh.edu (J.R.B.); 4 Department of Psychiatry and Behavioral Sciences, Johns Hopkins University School of Medicine, Baltimore, MD 21287, USA; tmoran@jhmi.edu; 5 Mallinckrodt Institute of Radiology, Departments of Radiology, Psychiatry, Neurology, Neuroscience, Washington University in St. Louis School of Medicine, St. Louis, MO 63110, USA; dfwong@wustl.edu

**Keywords:** caudate, high calorie, high fat, high sugar, ultra-processed, reward, positron emission tomography (PET), neurotransmitter, vertical sleeve gastrectomy (VSG), weight loss

## Abstract

The overconsumption of palatable energy-dense foods drives obesity, but few human studies have investigated dopamine (DA) release in response to the consumption of a palatable meal, a putative mediator of excess intake in obesity. We imaged [^11^C]raclopride in the brain with positron emission tomography (PET) to assess striatal dopamine (DA) receptor binding pre- and post-consumption of a highly palatable milkshake (250 mL, 420 kcal) in 11 females, 6 of whom had severe obesity, and 5 of whom had healthy-weight. Those with severe obesity underwent assessments pre- and 3 months post-vertical sleeve gastrectomy (VSG). Our results demonstrated decreased post- vs. pre-meal DA receptor binding in the ventral striatum (*p* = 0.032), posterior putamen (*p* = 0.012), and anterior caudate (*p* = 0.018), consistent with meal-stimulated DA release. Analysis of each group separately suggested that results in the caudate and putamen were disproportionately driven by meal-associated changes in the healthy-weight group. Baseline (pre-meal) DA receptor binding was lower in severe obesity than in the healthy-weight group. Baseline DA receptor binding and DA release did not change from pre- to post-surgery. The results of this small pilot study suggest that milkshake acutely stimulates DA release in the ventral and dorsal striatum. This phenomenon likely contributes to the overconsumption of highly palatable foods in the modern environment.

## 1. Introduction

Palatable energy-dense foods are ubiquitous in the modern food environment and widely acknowledged as a driver of obesity. Rodent models have demonstrated that meal-stimulated release of striatal dopamine (DA) may promote palatable food intake by both orosensory and post-ingestive pathways, providing a potential biological mechanism for overconsumption [1,2,3,4]. Further, alterations in DA signaling have been observed in both animal models and human studies of obesity. For example, consumption of a high-sugar, high-fat cafeteria diet in rodents leads to obesity and is accompanied by DA D2/D_3_ receptor downregulation and decreases in the ability of food and amphetamine to stimulate DA release in the nucleus accumbens (NAcc) [5,6,7]. Further, human functional magnetic resonance imaging (fMRI) studies suggest that striatal responses to food tastes are decreased with higher weight [8,9], supporting a hypothesis that depressed DA release may lead individuals with obesity to compensate by consuming more palatable foods to increase DA release to reach a reward threshold, and thereby increasing body weight (reward deficit hypothesis) [10,11].

Positron emission tomography (PET) imaging studies of human obesity have also demonstrated altered DA functioning, specifically altered striatal D_2_ receptor availability. However, the results have been mixed, and the outcome may depend on the body mass index (BMI) range, radioligand used, specific striatal sub-regions investigated, and interactions with pre-scan food intake [12]. For example, Wang et al. originally reported that the DA D_2_ receptor nondisplaceable binding potential (BP_ND_) [13] in the striatum, measured using [^11^C]raclopride, was decreased in participants with obesity [14], and this has been replicated in some subsequent studies [15,16,17], with null findings emerging from others [18,19,20]. Notably, Guo et al. [21], using [^18^F]fallypride in subjects spanning a broader BMI range, reported a negative relationship between ventral striatal D_2_ receptor availability and BMI but positive correlations in the dorsal and lateral striatum.

Bariatric surgery constitutes procedures to alter the integrity of the gastrointestinal tract to result in weight loss. The two most common bariatric interventions are the Roux-en-Y gastric bypass (RYGB) and the vertical sleeve gastrectomy (VSG). In both surgeries, the surgeon physically alters the normal integrity of the gastrointestinal tract, reducing the stomach to a fraction of its original size, from approximately 80 to 120 mL in VSG and approximately 20 to 30 mL in RYGB. Potential mechanisms for weight loss via these surgeries may include reduced gastric capacity and removal or reduction of the ghrelin-producing cells of the stomach, and increased postprandial anorexigenic gut peptide release, which contributes to decreased food intake and weight loss [22]. However, the impact of bariatric surgery at the level of the central nervous system is less understood.

The RYGB procedure may correct initially dysregulated striatal DA function. In a pilot PET study of bariatric surgery in humans, Steele et al. demonstrated increases in D_2_/D_3_ BP_ND_ of [^11^C] raclopride in the ventral striatum 6 weeks post-surgery in 4 out of 5 RYGB patients, with poor weight loss in the patient who did not demonstrate an increase D_2_/D_3_ BP_ND_ of [^11^C] raclopride in the ventral striatum [18]. In contrast, Dunn et al. [23] demonstrated widespread decreases in D_2_ availability 7 weeks post-surgery as indicated by decreases in [^18^F]fallypride BP_ND_ in response to RYGB and VSG in 5 subjects. The Steele et al. finding may therefore reflect increased D_2_ receptor availability in the ventral striatum, consistent with a rebound in reward circuitry accompanying the BMI reduction [18], and the Dunn finding decreased DA activity within the dorsal and lateral striatum [22]. However, both approaches demonstrated a return toward normal levels in response to surgery.

No studies of individuals with obesity, or individuals undergoing bariatric surgery, have used PET imaging to examine acute meal-stimulated DA release—an important putative mechanism driving consumption among individuals with both healthy weight and obesity and may be altered with surgical weight loss. The occurrence of palatable meal-induced DA in the dorsal putamen and caudate nucleus in healthy-weight controls is supported by data using a counter-balanced design taking place over two separate days [24]. Moreover, using a counter-balanced design over two separate days, Wang et al. [25] found that higher BMI was associated with evidence of lower DA release (i.e., higher DA BP_ND_) in the ventral striatum following a glucose vs. sucralose challenge. In contrast, Haltia et al. [26] observed no weight-related difference in DA release, notably using a design that compared a normal-weight group with an overweight-to-moderately obese, younger group who might be expected to show less evidence for dysregulated striatal DA function than a group exposed to severe obesity for a protracted period. As far as we are aware, DA release has not been demonstrated in humans both with and without obesity using an acute eating episode as we describe here.

Our primary aim for this small pilot study was to use [^11^C]raclopride PET to investigate DA release stimulated by a highly palatable meal among healthy-weight individuals and among those with severe obesity assessed pre- and post-bariatric surgery. To reduce the potential variability in brain dopamine responses by sex [27] and surgery type [28], as well as to reflect the majority (80%) population opting for weight loss surgery [29], we confined our study to adult females and females undergoing VSG. Since striatal sub-regions receive differential inputs and have differential functions [30], we examined five different regions in both the ventral and dorsal striatum. We hypothesized that the palatable meal would induce striatal DA release, especially in the ventral striatum (or NAcc), as measured by comparing pre-meal DA BP_ND_ to post-meal DA BP_ND_ across groups. As exploratory aims, we also investigated whether meal-stimulated DA release would be lesser in the participants with severe obesity as compared to those with healthy weight and whether meal-stimulated DA release would be altered post-surgery.

## 2. Materials and Methods

### 2.1. Participants

We recruited 5 females with healthy weight and 6 females with severe obesity undergoing VSG. Healthy-weight participants were recruited using flyers placed on the Johns Hopkins Medical Institutions (JHMI) East Baltimore and Johns Hopkins University (JHU) Homewood campuses. Surgery patients were recruited from the Johns Hopkins Center for Bariatric Surgery. For healthy-weight participants, an Institutional Review Board (IRB) approved telephone screening form was utilized to screen potential subjects for eligibility. Based on responses to the telephone screening, participants were invited to participate in an in-person screening assessment.

Inclusion criteria for healthy-weight and surgical participants were as follows: female non-smokers who were not pregnant or lactating; for healthy-weight females, further inclusion criteria were: age 18–40 years and in good health based on physical and psychological examination. For surgical patients, further inclusion criteria were: ages 18–55, BMI ≥ 35, weight ≤400 lbs (due to scanner bed constraints), medically and psychologically evaluated and approved for VSG surgery by the Johns Hopkins Center for Bariatric Surgery or any of the bariatric centers within the Johns Hopkins Clinical Research Network, and no clinically significant non-obesity related findings from a physical examination. Exclusion criteria for healthy-weight and surgical participants were: past or present clinically significant Axis I psychiatric diagnosis (including substance abuse), currently on psychoactive medication, lactose intolerance, currently experiencing a serious medical condition that would place them at risk or interfere with study participation, history of serious head injury with loss of consciousness for more than 10 min, contraindications for magnetic resonance imaging (MRI) (implanted metallic devices such as cardiac pacemaker or neurostimulator, some artificial joints or metal pins, surgical clips, other implanted metal parts, claustrophobia), history of syncope, poor peripheral venous access, radiation exposure in the past calendar year that in combination with the radiation exposure from this study would exceed 5 rem (effective dose), exposure to an investigational drug within 30 days, use of any prescription medications, over-the-counter or non-prescription preparations within 7 days prior to the scans unless deemed acceptable by the investigator. This study was approved by the Johns Hopkins Medical Institutions IRB (NA_00089426).

### 2.2. Procedures

We conducted PET scans with [^11^C]raclopride to measure DA BP_ND_ pre- and post-ingestion of a highly palatable milkshake in healthy-weight participants during a single assessment and in surgical patients 1–4 weeks pre- and 3 months post-surgery. All screening procedures and questionnaires were completed within 14 days prior to the PET scans. Before scanning, participants had a physical examination including measurements of weight and height as well as a complete medical and medication history, supine vital signs, 12-lead electrocardiogram (ECG), and laboratory safety tests (complete blood count (CBC) with a differential, comprehensive metabolic panel, urinalysis, urine toxicology screen, pregnancy test). Abnormal results or a positive pregnancy test triggered exclusion from the study.

#### 2.2.1. Questionnaire Measures

Surgical patients completed a battery of self-report questionnaires. These included the Food-Craving Inventory [FCI] [31] to assess cravings for foods classified as high fats (8 items; e.g., fried chicken, hot dog), sweets (8 items; e.g., ice cream, cookies), carbohydrates/starches (8 items; e.g., pancakes/waffles, pasta), and fast food (4 items; e.g., pizza, French fries), and the Behavioral Activation Scales [BAS] [32] to assess behavioral activation or approach, composed of sub-scales measuring pursuit of desired goals (Drive, e.g., I go out of my way to get things I want, 4 items), desire for new rewards and impulsive approach to potential rewards (Fun seeking, e.g., I crave excitement and new sensations, 4 items) and anticipation or occurrence of reward (Reward responsiveness, e.g., When I get something I want, I feel excited and energized, 4 items).

#### 2.2.2. Imaging Protocol

Participants presented to the PET imaging center and underwent a urine pregnancy test and a fasting glucose test via finger stick to ensure they were in the fasting state as instructed. An intravenous catheter was inserted into a vein of each arm, and then the participant was placed in the PET scanner. Following a 6–10 min transmission (attenuation) scan, participants were injected with ≈740 [megabequerels (MBqs)] [20 millicuries (mCis)] high-specific-activity [^11^C]raclopride, then underwent a pre-meal PET scan for the acquisition of dynamic PET data that lasted for 90 min in the 3D list mode. Following the pre-meal PET scan, participants were asked to drink 250 milliliters (mL) of Häagen-Daz vanilla bean milkshake (420 kcal) within 30 min (250 mL, 420 kiloCalories (kCal), 30 grams (g) fat, 44 g carbohydrates). The post-meal PET scan occurred 30 (±2 min) after completion of the test meal. Again, a 6- to 10-min transmission (attenuation) scan was performed, and participants underwent the PET scan after the [^11^C]raclopride injection. A structural MRI scan of the head was acquired for each participant for co-registration with the PET images and reviewed for incidental findings that would exclude them from PET scanning.

#### 2.2.3. Image Acquisition and Reconstruction 

High-resolution research tomography [HRRT], the currently highest resolution commercially-available brain-dedicated PET (Siemens/CPS), was used in order to maximize resolution (<2.5 mm spatial resolution) and increase the likelihood of detecting region-specific differences in BP_ND_. The emission scans were reconstructed [33], correcting for attenuation, scatter, and dead time. Each PET frame consisted of 256 by 256 by 207 (axial) cubic voxels of 1.22 mm. The final spatial resolution was about 2.3 mm in full width at half maximum in 3 directions [33]. The frame schedule was four 15-s, four 30-s, three 1-min, two 2-min, five 4-min, and twelve 5-min frames (a total of 30 frames in 90 min).

### 2.3. Statistical Analysis 

Small et al. [24] found evidence for palatable meal-induced [^11^C]raclopride DA release with an effect size of 0.5 using seven healthy-weight subjects. We, therefore, reasoned that *n* > 7 would give >60% power at α = 0.05 (two-tailed) to test our primary hypothesis of striatal DA release by a palatable milkshake across weight groups. We note that this represents an a priori power calculation that was conducted for the purposes of obtaining pilot data to support further work rather than for conducting a fully-powered analysis. Pre-meal BP_ND_ and post-meal BP_ND_ were estimated using the multilinear reference tissue method with 2 parameters (MRTM2) [34,35] for functional striatum subdivisions (5 per side) using the cerebellum as the reference region.

As described previously [36], the volumes of interest (VOIs) for putamen and caudate nucleus were defined on the SPGR MRI volumes using the threshold method of a locally developed VOI definition tool (VOILand). Then, the two VOIs were displaced in space such that the midline was vertical and the anterior-posterior commissure line was horizontal. On each coronal slice, the ventral striatum was defined as the portion of the striatal VOIs that were ventral to the line crossing the ventral corner of the lateral ventricle and perpendicular to the bisector of the internal capsule [37]. The remaining VOIs of the putamen and caudate nucleus were bisected by volume by vertical faces.

We used the definitions of functional striatum sub-divisions advanced by Martinez et al. [38] and Mawlawi et al. [39]. These represent realizations of in vitro connection studies by Haber et al. [40] and have been previously employed by our group [36]. This model has been supported by anatomical connectivity data obtained using diffusion-weighted MRI [41] and data comparing dopamine function between patients and controls by striatal division [42]. Based on these previous works, we believe that our anatomical guidelines divide the striatum into roughly functionally dissimilar subdivisions, e.g., the ventral striatum has greater anatomical and functional connections to the limbic system.

Our primary tests of meal-stimulated DA release were mixed models analysis of variance (ANOVA) with pre-meal BP_ND_ and post-meal BP_ND_ as a within-subjects factor and weight group (healthy-weight vs. severe obesity) as a between-subjects factor, implemented for each striatal subdivision (ventral striatum, anterior caudate, posterior caudate, anterior putamen, posterior putamen) using values averaged over the left and right hemispheres. For participants with severe obesity, we used data from the pre-surgery assessment only. These models were used to evaluate differences between pre-meal BP_ND_ and post-meal BP_ND_ across participants with healthy-weight and severe obesity (primary aim) and to test for differences in meal-stimulated DA release by weight group (exploratory aim 1). We also quantified DA release as (DARel) = (1 − BP_ND_ [post-meal]/BP_ND_ [pre-meal]), representing the percent reduction in D2/D3 BP_ND_ from pre-meal to post-meal, and conducted confirmatory independent *t*-tests comparing mean DARel values between groups for each striatal subdivision. To further explore the potential for weight group differences in DA release, we additionally ran post-hoc paired *t*-tests comparing pre-meal BP_ND_ and post-meal BP_ND_ in the healthy-weight and severe obesity groups separately. Moreover, to test whether reduced baseline BP_ND_ assessed pre-meal could have contributed to group differences or lack thereof in DA release, we ran post-hoc one-tailed independent *t*-tests comparing pre-meal BP_ND_ between groups for each striatal subdivision.

Finally, to test for changes in meal-stimulated DA release from pre- to post-surgery in the severe obesity group (exploratory aim 2), we ran mixed models ANOVAs with pre-meal BP_ND_ vs. post-meal BP_ND_ as one within-subjects factor and pre-surgery vs. post-surgery as a second within-subjects factor. We evaluated interactions between meal conditions and surgery conditions. We also ran confirmatory paired *t*-tests comparing mean DARel values from pre- to post-surgery for each striatal subdivision.

## 3. Results

### 3.1. Participant Characteristics

Participant characteristics, including BMI and questionnaire data (surgical patients only), are in Table 1. All participants were female. Healthy-weight participants (*n* = 5) had a mean age of 24.0 [standard deviation (SD) 4.18] and a mean BMI of 21.78 (0.16), with *n* = 1 describing themselves as white, *n* = 1 as African-American or Black, and *n* = 3 as Asian. Surgical patients (*n* = 6) had a mean age of 43.5 (SD 8.0) and a mean BMI of 41.8 (5.5) at the pre-surgery assessment and 34.8 (4.2) at the post-surgery assessment, with *n* = 2 describing themselves as white, and *n* = 5 as African-American or Black. Age differed by weight group (t = −4.88, *p* < 0.001), and BMI differed by weight group in both pre-surgery (t = −8.08, *p* < 0.001) and post-surgery (t = −6.82, *p* < 0.001). From pre- to post-surgery, the BMI decreased (t = 10.103, *p* < 0.001, *n* = 6), FCI carbohydrates scores decreased (t = 3.302, *p* = 0.030, *n* = 5), and BAS Drive scores increased (t = −3.5, *p* = 0.025, *n* = 5).

### 3.2. Meal-Stimulated Change in DA BP_ND_ in the Healthy-Weight Group and Severe Obesity Group Pre-Surgery

**Figure 1:**
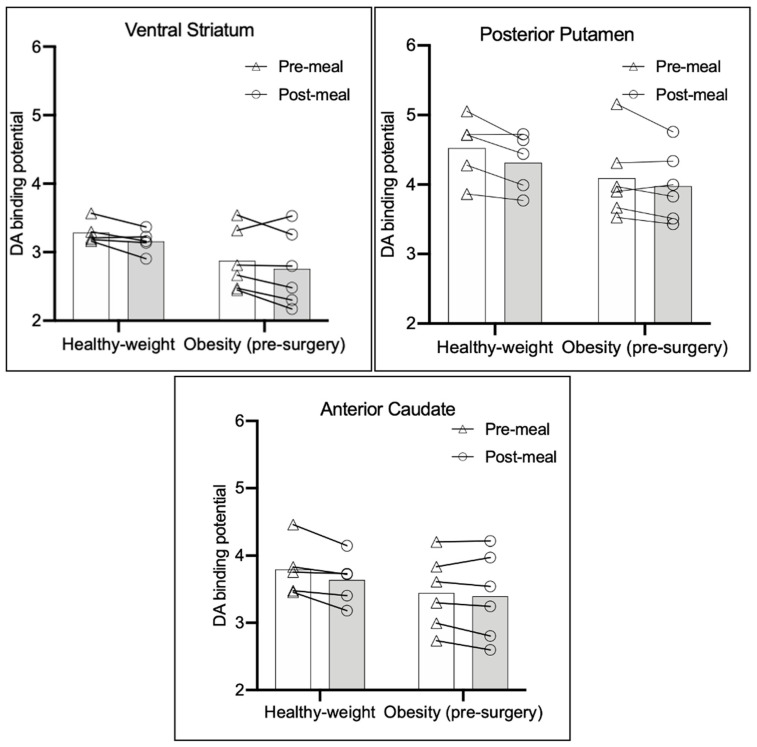
Group means and individual pre-meal and post-meal BP_ND_ values for ventral striatum, posterior putamen, and anterior caudate for the healthy-weight group and severe obesity group at the pre-surgery assessment. Bars represent group means, and lines represent values for individuals. Pre-meal to post-meal decreases were apparent across both groups for each region depicted (all *p* < 0.05). Pre-meal values in the ventral striatum were lower in the severe obesity vs. healthy-weight group (one-tailed *p* < 0.05).

The group means and individual values for pre-meal and post-meal BP_ND_ in the healthy-weight group and the severe obesity group at the pre-surgery assessment are presented in Figure 1 (regions showing significant meal-stimulated changes only) and Supplementary Figure S1 (results for all regions). The mean DARel values are presented in Supplementary Table S1.

Mixed models ANOVAs combining data from healthy-weight participants, and pre-surgery data from participants with severe obesity, were used to test for differences in BP_ND_ from pre-meal to post-meal across groups (primary aim) and differences in meal-stimulated DA release between groups (exploratory aim 1). Pre-meal to post-meal decreases were found in the ventral striatum (F = 6.44 *df* = 9 *p* = 0.032), posterior putamen (F = 9.84 *df* = 9 *p* = 0.012), and anterior caudate (F = 8.27 *df* = 9 *p* = 0.018). No time × group interactions were apparent, and, consistent with these results, confirmatory independent *t*-tests comparing the mean DARel values between groups for each striatal subdivision revealed no group differences. However, paired *t*-tests conducted separately for each weight group demonstrated significant meal-stimulated decreases in the anterior putamen (t = 2.98 *df* = 4 *p* = 0.041), posterior putamen (t = 2.8 *df* = 4 *p* = 0.048), and anterior caudate (t=2.79 *df* = 4 *p* = 0.049) for healthy-weight participants, with no significant changes in any region emerging for surgery patients. Additionally, one-tailed independent *t*-tests comparing pre-meal BP_ND_ between groups for each striatal subdivision found that pre-meal BP_ND_ in the ventral striatum was significantly reduced in the severe obesity vs. healthy-weight group (t = 1.9 *df* = 9 *p* = 0.046). We note that none of these findings withstood Bonferroni correction for multiple comparisons across the five striatal subdivisions tested.

### 3.3. Meal-Stimulated Change in DA BP_ND_ in the Severe Obesity Group from Pre- to Post-Surgery

The group means and individual values for pre-meal and post-meal BP_ND_ for the severe obesity group at both the pre- and post-surgery assessments for each striatal subdivision are presented in Supplementary Figure S1, with the mean DARel values in Supplementary Table S1.

Mixed models ANOVAs with pre-meal BP_ND_ vs. post-meal BP_ND_ as one within-subjects factor and pre-surgery vs. post-surgery as a second within-subjects factor were conducted to investigate differences in meal-stimulated DA release from pre to post-surgery (exploratory aim 2). They revealed no interactions between meal conditions and surgery conditions. Consistent with these results, confirmatory paired *t*-tests comparing the mean DARel values from pre- to post-surgery for each striatal subdivision also revealed no differences.

## 4. Discussion

We here report pilot data collected in a small sample on meal-stimulated DA release, operationalized as the change in binding potential as endogenously released DA, stimulated by a palatable high-sugar, high-fat liquid meal, competes with the radiotracer [^11^C]raclopride for D_2_ receptors. Our results should be treated with caution due to the small sample size but are consistent with the occurrence of intrasynaptic DA release (also known as phasic release) in both the ventral and dorsal striatum, both of which play a vital role in food reward [43,44,45]. Notably, the effects that we detected across groups occurred in the posterior portion of the putamen, which plays a role in sensorimotor processing, and the anterior portion of the caudate (caudate head), which plays a role in cognition and emotion, including the valuation of action [30]. DA release in these regions may therefore contribute to food reward and food seeking and drive the overconsumption of high-sugar, high-fat foods in the modern environment. While the magnitude of the binding changes may appear small, they should be viewed in the context of prior results. Amphetamine administration (0.3 mg/kg) has been demonstrated to result in a 10–20% decrease in striatal D_2_ binding [46,47,48,49,50]. Thus decreasing radiotracer binding by 3–5%, as demonstrated here, represents a meaningful change in DA release. Our results add to previous literature documenting DA release in response to ingestion of a favorite meal [22], ingestion of a 75 g glucose drink [25], 300 mg/kg intravenous glucose infusion [26], and ingestion of small tastes of milkshake [51] by demonstrating that DA release is acutely stimulated by ingestion of a regular portion of high-fat high-sugar milkshake (250 mL, 420 kcal).

Notably, whereas previous studies included a mix of both males and females [14,24,26] or males only [51], our study was conducted exclusively in females, enabling us to draw conclusions specifically about a population that is afflicted disproportionately by severe obesity [52] and binge eating [53], and presents more frequently for bariatric surgery [29].

In addition, our pilot study extends the findings of the study employing [^11^C]raclopride to examine DA responses to milkshake tastes in healthy-weight males by reporting meal-simulated DA release across females with both healthy-weight and severe obesity. Our findings are significant given that recent and older studies have demonstrated a supra-additive effect of combining fat and carbohydrate in high quantities on the neural processing of food reward [54]. A valuable next step to test this hypothesis would be to investigate DA release to high sugar vs. high fat vs. high sugar+fat meals in adults with healthy-weight and severe obesity.

Our small sample size did not allow a rigorous test of our secondary aim to investigate weight group differences in DA release. However, it was notable that findings in the caudate and putamen, rather than in the ventral striatum, were significant when confining the analysis to healthy-weight individuals. Classic experiments demonstrate that midbrain DA neurons increase their firing when they encounter a reward, but that over time this increased firing begins to be triggered by cues predicting the reward [55], with DA neurons throughout the striatum responding to both the primary food reward and reward-predictive cues in a region-specific manner [56]. This is consistent with PET data demonstrating increased occupancy of DA receptors in the striatum during exposure to cocaine craving cues in the absence of pharmacological challenge [57]. Further, a number of human fMRI studies support that individuals at risk for obesity initially demonstrate heightened striatal responses to food tastes that decrease with weight gain [10]. Since a recent [^11^C]raclopride study of healthy-weight males examining activation to milkshake taste by time bins revealed early orosensory-stimulated activation of NAcc followed by later activation of the caudate head [51], it is possible that our findings reflect a stronger relative post-ingestive DA response in females with healthy-weight than those with severe obesity. It would therefore be valuable to directly investigate in a larger sample whether the relative activation of different striatal regions over time differs by weight group.

A potential reason for our failure to observe changes in the DA receptor binding or release post-surgery in this small pilot study may have been that all participants underwent VSG rather than RYGB. Although the current sample demonstrated substantive weight loss 3 months post-surgery (decrease of 7.02 BMI points), in a larger, overlapping sample, we found that VSG produced relatively less weight loss (6.6 BMI points for VSG vs. 7.74 BMI points for RYGB) as well as evidence for less of an impact on brain reward circuits and food motivation [28,58]. One possible mechanism for differences between surgery types in effects on food intake and weight loss may be that the transection of the proximal jejunum, attachment of the Roux limb to the gastric pouch, and reattachment of the stomach, duodenum, and proximal jejunum to the distal jejunum in the case of RYGB causes rapid delivery of nutrients to the distal small intestine producing larger initial alterations in satiety-promoting hormones PYY and GLP-1 [59]. PYY infusion [60] and GLP-1 receptor activation [61] have been shown to influence the activation of brain reward regions, including the caudate and putamen, in response to food cues, while rodent data support that the inhibition of food intake via GLP-1 receptor activation is mediated in part by mesolimbic reward system structures including nucleus accumbens [62]. Potential differences between surgery types on brain dopamine responses could thus be mediated by differences in gut hormone release. However, they could also be mediated indirectly by effects of these or other changes on food intake, i.e., larger reductions in consumption of high sugar, high-fat foods in the case of RYGB may allow normalization of previous down-regulation of DA receptors [5,6,7,10,11].

Descriptive questionnaire data collected among the surgery patients supported a decrease in cravings for high carbohydrate foods as assessed by the FCI and an increase in the pursuit of desired goals as assessed by the BAS Drive sub-scale. Such behavioral results in this small sample may not be considered definitive. However, the evidence for a decrease in food cravings is consistent with results at 6 months and 1 year post-surgery that we reported in a larger, overlapping sample, which demonstrated decreases in total food cravings as well as cravings for sweets, fats, carbohydrates, and fast food [58]. The evidence for increased motivation is also consistent with studies demonstrating decreases in depression following bariatric surgery [63]. Larger studies may test whether individual variation in DA function changes post-surgery might explain individual variation in post-surgical changes in food cravings and in post-surgical increases in general motivation, which the dopamine system may also mediate [64].

Limitations of this small pilot study include several factors that may reduce the reliability of the findings we report. These include the unbalanced age and race distributions between groups, which could not be controlled for due to non-overlapping values in this small sample. Previous research has demonstrated decreased DA D_2_/D_3_ receptor binding with older age [65], and we cannot rule out the possibility that the reduced baseline binding in the ventral striatum that we observed in the severe obesity group could be due to their older age. In addition, our study did not control for menstrual status, our design was unable to distinguish between the contributions of oral stimulation vs. post-ingestive nutrient sensing, and we did not conduct analyses of glucose and insulin levels to inform on the role of metabolic contributions to striatal DA release in response to food stimulation. These are valuable directions that warrant a more detailed investigation [66].

Future directions to build on this small study could include replications with the addition of a tasteless solution and using a larger sample obtained at multiple centers supplying adequate groups of age- and race-matched participants with obesity and healthy weight. Such studies could further elucidate the functional consequences of observed differences in DA release by including analyses of correlations between behavioral data and neuroimaging results. Larger studies would also support voxel-wise analyses within the striatum to provide more granular information on the location of DA release.

## Figures and Tables

**Table 1:** Participant characteristics.

	Healthy-Weight (*n* = 5)	Severe Obesity (*n* = 6)	
		Pre-Surgery	Post-Surgery
Age	24.0 ± 4.18 ^†^	43.5 ± 8.02 ^†^	
Race (*n* Black; *n* White; *n* Asian)	1; 1; 3	4; 2; 0	
BMI	21.8 ± 0.16 ^†^	41.8 ± 5.48 ^†,^*	34.78 ± 4.22 ^†,^*
Food-Craving Inventory (FCI)		(*n* = 5)	(*n* = 5)
- Sweets	-	1.78 ± 0.67	1.23 ± 0.18
- Fats	-	1.85 ± 0.54	1.44 ± 0.14
- Carbohydrates	-	1.88 ± 0.60 *	1.32 ± 0.33 *
- Fast food	-	2.15 ± 0.76	2.10 ± 0.38
Behavioral Activation Scales (BAS)		(*n* = 5)	(*n* = 5)
- Reward responsiveness	-	3.52 ± 0.33	3.48 ± 0.33
- Drive	*-*	2.75 ± 0.53 *	3.10 ± 0.38 *
- Fun-seeking	*-*	3.15 ± 0.22	3.10 ± 0.60

Values are mean (SD) unless otherwise specified. *n* per analysis is at the top of the column unless otherwise specified. ^†^ = significantly different between the healthy-weight group and severe obesity group pre-surgery, *p* < 0.05 * = significantly different from pre- to post-surgery *p* < 0.05.

## Data Availability

Data are available from the corresponding author on request.

## Supplementary Materials

The following supporting information can be downloaded at: https://www.mdpi.com/article/10.3390/nu15122671/s1, Figure S1: Group means and individual pre-meal and post-meal BP_ND_ values for all striatal subdivisions for the healthy-weight group and severe obesity group at the pre- and post-surgery assessments. Table S1: Mean ± SD DARel values for all striatal subdivisions for the healthy-weight group and severe obesity group at the pre- and post-surgery assessments.

## Abbreviations

ANOVA	Analysis of variance
BAS	Behavioral Activation Scales
BMI	Body mass index
BP_ND_	Nondisplaceable binding potential
CBC	Complete blood count
DA	Dopamine
DARel	Dopamine release
ECG	Electrocardiogram
FCI	Food-Craving Inventory
fMRI	Functional magnetic resonance imaging
g	Gram
HRRT	High-Resolution Research Tomography
IRB	Institutional Review Board
JHMI	Johns Hopkins Medical Institutions
JHU	Johns Hopkins University
kCal	kilocalorie
MBqs	Megabequerels (MBqs
mCis	Millicuries (mCis)
mL	Milliliters
MRI	Magnetic resonance imaging
MRTM2	Multilinear reference tissue method with 2 parameters
N	Number
NAcc	Nucleus accumbens
PET	Positron emission tomography
RYGB	Roux-en-Y gastric bypass
SD	Standard deviation
VSG	Vertical sleeve gastrectomy
