# Supplementary material for: Milkshake Acutely Stimulates Dopamine Release in Ventral and Dorsal Striatum in Healthy-Weight Individuals and Patients with Severe Obesity Undergoing Bariatric Surgery: A Pilot Study
Source: Nutrients. 2023 Jun 8;15(12):2671. doi: 10.3390/nu15122671 (PMC10302648; doi:10.3390/nu15122671)
Supplement: Supplementary file 1 [file nutrients-15-02671-s001.zip › nutrients-2396685-supplementary.pdf]

Milkshake acutely stimulates dopamine release in ventral and dorsal striatum in healthy-weight individuals and patients with severe obesity undergoing bariatric surgery: a pilot study

Figure S1. Group mean and individual pre-meal and post-meal BP_{ND} values for all striatal subdivisions for the healthy-weight group and severe obesity group at the pre- and post-surgery assessments.

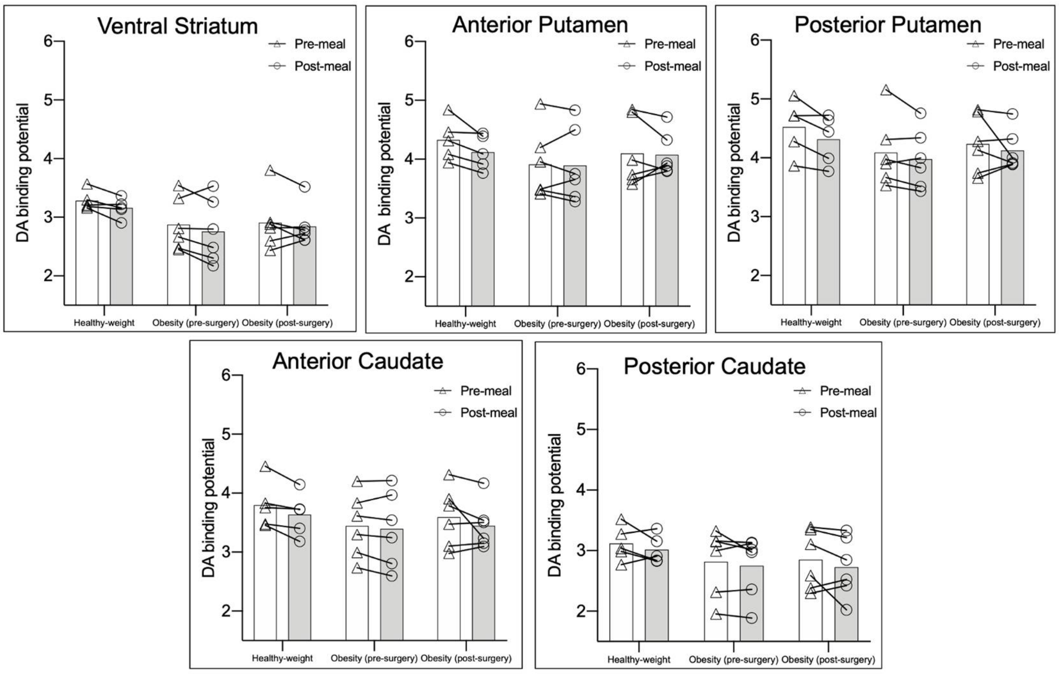

***Table S1:** Mean+SD DARel values for all striatal subdivisions for the healthy-weight group and severe obesity group at the pre- and post-surgery assessments*

	Healthy-weight (n=5)	Severe obesity (n=6)	
		Pre-surgery	Post-surgery
Ventral striatum	3.7 ± 3.4	4.5 ± 6.4	1.5 ± 6.8
Anterior caudate	4.1 ± 3.2	1.9 ± 3.6	3.5 ± 7.7
Posterior caudate	2.9 ± 6.5	2.1 ± 5.2	4.1 ± 10.3
Anterior putamen	4.7 ± 3.2	0.4 ± 5.1	-0.1 ± 7.1
Posterior putamen	4.5 ± 3.4	2.0 ± 8.2	2.6 ± 3.6
